# Robust Universal Inference

**DOI:** 10.3390/e23060773

**Published:** 2021-06-18

**Authors:** Amichai Painsky, Meir Feder

**Affiliations:** 1 The Industrial Engineering Department, Tel Aviv University, Tel Aviv 6997801, Israel; 2 The School of Electrical Engineering, Tel Aviv University, Tel Aviv 6997801, Israel; meir@eng.tau.ac.il

**Keywords:** minimax estimation, minimax risk, statistical inference, estimation theory, universal prediction

## Abstract

Learning and making inference from a finite set of samples are among the fundamental problems in science. In most popular applications, the paradigmatic approach is to seek a model that best explains the data. This approach has many desirable properties when the number of samples is large. However, in many practical setups, data acquisition is costly and only a limited number of samples is available. In this work, we study an alternative approach for this challenging setup. Our framework suggests that the role of the train-set is not to provide a single estimated model, which may be inaccurate due to the limited number of samples. Instead, we define a class of “reasonable” models. Then, the worst-case performance in the class is controlled by a minimax estimator with respect to it. Further, we introduce a robust estimation scheme that provides minimax guarantees, also for the case where the true model is not a member of the model class. Our results draw important connections to universal prediction, the redundancy-capacity theorem, and channel capacity theory. We demonstrate our suggested scheme in different setups, showing a significant improvement in worst-case performance over currently known alternatives.

## 1. Introduction

One of the major challenges in statistics and machine learning is making predictions and inference from a limited number of samples. This problem is mostly evident in modern statistics (big data), where the dimension of the problem is very large compared to the number of samples in hand, or in cases where data acquisition is relatively costly, and only a small number of samples is available (such as in complicated clinical trails). The standard approach in many applications is to seek a model that best explains the data. For example, empirical risk minimization (ERM) [1] is a commonly used criterion in predictive modeling. Minimizing the empirical risk has many desirable properties. Under different loss functions, we may attain consistency, unbiasedness, and other favorable attributes. In parametric estimation, perhaps the most popular approach is maximum likelihood. Here, again, we seek parameters that maximize the likelihood of the given set of observations.

However, what happens if our specific instance of data does not represent the true model well enough (as happens in high-dimensional problems)? Is it still desirable to choose the single model that best explains it?

In this work, we study an alternative approach for this challenging setup. Here, instead of choosing a model that best describes the data, we define a class of models that describe it with high confidence. Then, we seek a scheme that minimizes the worst-case loss in the class. This way, we control the performance over a class of reasonable models and provide explicit worst-case guarantees, even when the given data fail to accurately represent the true model. This scheme is, in fact, a data-driven approach of minimax estimation, as later discussed. Further, we show it provides worst-case guarantees for the expected regret of future samples. This property makes our framework applicable both for inference and prediction tasks.

One of the major challenges of our suggested scheme is to characterize the model class. In this work, we consider a class of models that corresponds to a confidence region of the unknown parameters. This way we provide a PAC-like generalization bound, as the true model is a member of this class with high confidence. Then, we introduce a more robust approach which drops the model class assumptions and provides stronger performance guarantees for the derived estimator. We demonstrate our suggested approach in classical inference problems and more challenging large alphabet probability estimation. We further study a real-world example, motivated from the medical domain. Our suggested approach introduces favorable worst-case performance, for every given instance of data, at a typically low cost on the average. This demonstrates an “insurance-like” trade-off; we pay a small cost on the average to avoid a great loss if “something bad happens” (that is, the observed samples do not represent the true model well enough).

The rest of this manuscript is organized as follows. In Section 2, we review related work to our problem. We introduce our suggested framework and some of its basic properties in Section 3. Then, we extend the framework to a more robust estimation scheme in Section 4. We demonstrate our suggested scheme in several setups. In Section 5, we study the unknown normal mean problem, while in Section 6 we focus on multinomial probability estimation. We consider a more challenging large alphabet probability estimation problem in Section 7. Finally, we study a real-work breast cancer problem in Section 8. We conclude with a discussion in Section 9.

## 2. Previous Work

Minimax estimation has been extensively studied over the years. Here, we briefly review the more relevant results for our work. Let $x^{n}∼p_{\theta }^{n}$ be a collection of *n* i.i.d. samples, drawn from a distribution $p_{\theta }$, where θ is a fixed and unknown parameter. Let Θ be a given class of parameters. Assume that θ∈Θ. Let $\hat{\theta }≜\hat{\theta }\left(x^{n}\right)$ be an estimator of θ from $x^{n}$. Let $R(\theta ,\hat{\theta })$ be a risk function which measures the expected error between the true parameter θ and its corresponding estimate $\hat{\theta }$. For example, $R\left(\theta ,\hat{\theta }\right)=E_{x^{n}∼p_{\theta }^{n}}{\left(\theta -\hat{\theta }\left(x^{n}\right)\right)}^{2}$ is the mean squared error. The minimax risk [2] is defined as

(1) $$r_{mm}=\underset{\hat{\theta }}{\inf}\underset{\theta \in \Theta }{\sup}R\left(\theta ,\hat{\theta }\right).$$

A minimax estimator ${\hat{\theta }}_{mm}$ satisfies $\sup_{\theta \in \Theta }R\left(\theta ,{\hat{\theta }}_{mm}\right)=r_{mm}$, if such exists. In words, ${\hat{\theta }}_{mm}$ minimizes the worst-case risk for a given class of parameters Θ. Finding the minimax estimator is, in general, not an easy task. However, the optimal solution is characterized by several important properties.

Let ${\hat{\theta }}_{\pi }=\int_{\theta \in \Theta }\theta \pi \left(\theta \right)d\theta$ be a Bayes estimator with respect to some prior π(θ) over Θ. In words, ${\hat{\theta }}_{\pi }$ is a weighted average of θ∈Θ, according to a given weight function π≜π(θ). Let $r_{\pi }=\int_{\theta \in \Theta }R\left(\theta ,{\hat{\theta }}_{\pi }\right)d\pi \left(\theta \right)$ be the average risk with respect to π. One of the basic results in the minimax theory suggests that if $r_{\pi }=r_{mm}$, then ${\hat{\theta }}_{\pi }$ is a minimax estimator and π is a least favorable prior (satisfying $r_{\pi }\geq r_{\pi^{\prime }}$ for any $\pi^{\prime }$) [2]. Importantly, if a Bayes estimator has a constant risk, it is minimax. Note that this is not a necessary condition.

For example, consider the problem of estimating the mean of a *d*-dimensional Gaussian vector. Here, it can be shown that the maximum likelihood estimator (MLE) is also the minimax estimator with respect to the squared error. Interestingly, in this example, the MLE is known to be inadmissible for d2; assuming that the mean is finite, the famous James–Stein estimator [3] dominates the MLE, as it achieves a lower mean squared error (where the phenomenon is more evident as the mean is closer to zero) [2,3]. Additional examples for the minimax estimators are provided in [2].

The minimax formulation was studied in a variety of setups. In [4], the authors considered minimax estimation of parameters over $L^{p}$ loss and provided key analytical results. These results were further studied and generalized (for example, see in [5]). Bickel studied minimax estimation of the normal mean when the parameter space is restricted [6]. Later, Marchand and Perron considered the case where the norm of the normal mean is bounded [7]. The minimax approach is also applicable to supervised learning problems. In [8], the authors considered minimax classification with fixed first- and second-order moments. Eban et al. developed a classification approach by minimizing the worst-case hinge loss subject to fixed low-order marginals [9]. Razaviyayn et al. fitted a model that minimizes the maximal correlation under fixed pairwise marginals to design a robust classification scheme [10]. Farnia et al. described a minimax approach for supervised learning by generalizing the maximum entropy principle [11].

The minimax approach has many applications, as it defines a conservative estimate for a given class of models. A variety of examples spans different fields including optimization [12], signal processing [13,14], communications [15], and others [16].

It is important to emphasize that the minimax problem (1), and its corresponding solution, heavily depend on the assumption that the unknown parameter θ is a member of the given class of parameters Θ. However, what happens if this assumption is false, and θ∉Θ (as discussed, for example, in [17,18,19])? Furthermore, how do we choose Θ in practice? If we choose Θ to be too large, we might control a class of models that are unreasonable. On the other hand, if Θ is too small, we may violate the assumption that θ∈Θ. Finally, notice that the minimax problem is typically concerned with the expected worst-case performance (the risk). However, in many real-world applications we are given a single instance of data, which may be quite costly to acquire. Therefore, we require worst-case performance guarantees for this specific instance of data. In this work, we address these concerns and suggest a robust, data-driven, universal estimation scheme for a given set of observations.

## 3. The Suggested Inference Scheme

For the purpose of our presentation, we use the following additional notations. Let $\Theta_{r}$ be a *restricted* class of parameters and denote $P(\Theta_{r})$ as the corresponding *restricted* class of parametric distributions. Assume that the true model $p_{\theta }$ is a member of $P(\Theta_{r})$ (or alternatively, $\theta \in \Theta_{r}$). For example, $p_{\theta }=N\left(\theta ,1\right)$ is a normal distribution with an unknown mean θ and a unit variance, while $P(\Theta_{r})$ is a set of all normal distributions with $\theta \in \Theta_{r}=\left[\theta_{a},\theta_{b}\right]$ (henceforth, restricted to $\left[\theta_{a},\theta_{b}\right]$) and a unit variance. Let P={p|p(x)≥0,∫p(x)dx=1} be the class of all probability measures. Let $q≜q(\cdot |x^{n})$ be a probability measure which estimates $p_{\theta }$ given the samples $x^{n}$. Notice that as opposed to the presentation in (1), the estimator *q* is with respect to the entire probability distribution $p_{\theta }$, and not just unknown parameter θ. We measure the estimate’s accuracy using the Kullback–Leibler (KL) divergence between the true underlying distribution $p_{\theta }$ and *q*, formally defined as $D_{\mathrm{KL}}(p_{\theta }\left||q\right)=\int p_{\theta }\left(x\right)\log\frac{p_{\theta }\left(x\right)}{q(x)}dx$. The KL divergence is a widely used measure for the discrepancy between two probability distributions, with many desirable properties [20]. In addition, the KL divergence serves as an upper bound for a collection of popular discrepancy measures (for example, the Pinsker inequality [21] and the universality results in [22,23]). In this sense, by minimizing the KL divergence, we implicity bound from above a large set of common performance merits.

Ultimately, our goal is to find an estimate *q* that minimizes $D_{\mathrm{KL}}(p_{\theta }\left||q\right)$ for the unknown θ. Thus, we consider a minimax formulation

(2) $$\begin{array}{c} \min_{q}\underset{p_{\theta }\in P\left(\Theta_{r}\right)}{\sup}D_{\mathrm{KL}}(p_{\theta }\left||q\right) \end{array}$$

where q∈P is the minimizer of the worst-case divergence over the class $P(\Theta_{r})$, if such exists. In words, *q* minimizes the worst possible divergence, over the restricted model class $P(\Theta_{r})$. To avoid an overload of notation, we assume that q∈P throughout the text, unless otherwise stated. We observe several differences between (1) and (2). First, the formulation in (1) considers an estimate $\hat{\theta }$, and by that implicitly restricts the solution to be a parametric distribution $p_{\hat{\theta }}$ of the same family as $p_{\theta }$. On the other hand, (2) considers the entire distribution and does not impose any restrictions on the solution. Second, the standard minimax formulation (1) focuses on the risk. Our approach considers the estimation accuracy for every given instance of data ($q≜q(\cdot |x^{n})$, as defined above). Third, notice that $D_{\mathrm{KL}}(p_{\theta }\left||q\right)$ can also be viewed as the expected log-loss regret, where the expectation is with respect to a future sample, $D_{\mathrm{KL}}(p_{\theta }\left||q\right)=E_{x∼p_{\theta }}\left(l\left(x,q\right)-l\left(x,p_{\theta }\right)\right)$ and l(x,q)=−log(q(x)) is the logarithmic loss. This means that while (1) focuses on the expected loss with respect to the given samples, (2) considers the expected performance of future samples. We further discussed these points in Section 5, Section 6 and Section 8.

In practice, one of the major challenges in applying any minimax formulation is the choice (or design) of the parametric class. Specifically, using the notations of (2), choosing a larger class $\Theta_{r}$ is more likely to include the true model θ, but may also include unreasonable worst-case models (for example, $\Theta_{r}=R$ in the unknown normal mean example above). On the other hand, choosing a more restrictive $\Theta_{r}$ may violate our assumption that $\theta \in \Theta_{r}$. Therefore, a trade-off between the two seems inevitable. In the following, we focus on the design and characterization of a set $\Theta_{r}$ that depends on the given samples, $\Theta_{r}\left(x^{n}\right)$. In other words, we use the train-set $x^{n}$ to construct a minimal-size restricted model class $\Theta_{r}\left(x^{n}\right)$ that contains the true parameter θ with high confidence. Then, we solve the minimax problem (2) with respect to it and attain an estimator that minimizes the maximal divergence in the class (or equivalently, the expected log-loss regret for future samples).

### Designing and Controlling the Restricted Model Class

Our first objective is to construct a minimal-size $\Theta_{r}\left(x^{n}\right)$ such that $\theta \in \Theta_{r}\left(x^{n}\right)$ with high confidence. For this purpose, we turn to classical statistics and construct a confidence region for the desired parameter θ. A confidence region of level 100(1−α)% is defined as a region T such that P(θ∈T)=1−α. Notice that T is random and depends on the samples $x^{n}$, while θ is an unknown (non-random) parameter. Further, notice that a confidence region T is data-dependent and does not require knowledge of the true parameter θ. Obviously, there are many ways to define T to satisfy the above. We are interested in a confidence region that has a minimal expected volume. For example, consider *n* i.i.d. samples from N(θ,1), as discussed above. Let $\bar{x}$ be the sample mean. Then, the minimal-size 100(1−α)% confidence interval is $\left[\bar{x}\pm z_{\frac{\alpha }{2}}\frac{1}{\sqrt{n}}\right]$, where $z_{\alpha }$ is the upper 100α percentile of a standard normal distribution [24].

Given the restrictive model class, we would like to solve the minimax problem defined in (2). A general form of this problem was extensively studied over the years, mainly in the context of universal compression and universal prediction [15]. There, $D_{\mathrm{KL}}(p_{\theta }\left||q\right)$ is the expected number of extra bits (over the optimal code-book), required to code samples from $p_{\theta }$ using a code designed for *q*. The celebrated redundancy-capacity theorem demonstrates a basic connection between the desired formulation (2) and channel capacity theory. Let T∼π be a source variable, *X* be a target variable and $P(\Theta_{r})$ be the set of transition probabilities from *X* to *T*. In other words, *T* is a message, transmitted through a noisy channel, characterized by $P(\Theta_{r})$. The received (noisy) message is denoted by *X*. Let I(T;X) be the mutual information between *T* and *X*, and $C\left(\Theta_{r}\right)≜\sup_{\pi }I\left(T;X\right)$ be the corresponding channel capacity. The redundancy-capacity theorem [25,26,27] suggests that for $C(\Theta_{r})\infty$, the minimax formulation presented in (2) is equivalent to

(3) $$\begin{array}{c} \underset{\pi (\theta )}{\sup}\int_{\theta \in \Theta_{r}}\pi \left(\theta \right)D_{\mathrm{KL}}(p_{\theta }\left|\right|q_{\pi })d\theta =\underset{\pi }{\sup}I\left(T;X\right)≜C\left(\Theta_{r}\right) \end{array}$$

where π(θ) is a weight function for every $\theta \in \Theta_{r}$ and $q_{\pi }=\int_{\theta \in \Theta_{r}}\pi \left(\theta \right)p_{\theta }d\theta$ is a *mixture distribution*. In words, solving (2) is equivalent to solving a channel capacity problem. Furthermore, the source distribution which maximizes the mutual information between the source and the target (and henceforth achieves the channel capacity) is a mixture over $P(\Theta_{r})$. This solution is quite similar to the solution of (1); in both cases, we obtain a Bayes estimator over the given class, while the least favorable prior (or equivalently, the capacity achieving prior), if such exists, attains the maximal average risk (the channel capacity). See examples in Section 5 and Section 6 for further detail. It is important to emphasize that while θ is a fixed and unknown parameter, π(θ) is a weight function for every $\theta \in \Theta_{r}$, and $q_{\pi }$ is a weighted average over $P(\Theta_{r})$.

The redundancy-capacity theorem shows that the solution to the minimax problem (2) is achieved by solving the channel capacity problem (3). We apply the capacity-redundancy theorem to our suggested class $\Theta_{r}\left(x^{n}\right)$ to attain the desired solution. Theorem 1 below summarizes our parametric inference approach.

**Theorem** **1.** *Let $x^{n}∼p_{\theta }^{n}$ be a collection of n i.i.d. samples, drawn from an unknown distribution $p_{\theta }$. Let $\Theta_{r}\left(x^{n}\right)$ be a 100(1−α)% confidence region for the parameter θ. Assume that $C(\Theta_{r}\left(x^{n}\right))\infty$. Then, with probability 1−α (over the samples), $C(\Theta_{r}\left(x^{n}\right))$ is the minimal worst-case divergence and $q_{\pi }$ is the corresponding minimax estimator, denoted as the mixture model.*

Theorem 1 establishes a PAC-like generalization bound for parametric inference. It defines the worst-case expected performance of future samples (with respect to a logarithmic loss, as discussed above), at a confidence level of 1−α over the drawn samples. Specifically, with probability 1−α we have that $E_{x∼p_{\theta }}\left(l\left(x,q_{\pi }\right)-l\left(x,p_{\theta }\right)\right)\leq C\left(\Theta_{r}\left(x^{n}\right)\right)$ for the entire parametric class. It is important to emphasize that the resulting minimax estimator $q_{\pi }$ is data-dependent, as it is a mixture over the data-driven restricted model class.

Solving the channel capacity problem is, in general, not an easy task. However, there exist several cases where the solution to (3) holds a closed-form expression, or an efficient computational routine. We demonstrate basic examples in Section 5 and Section 6.

## 4. A Generalized Inference Scheme beyond the Restricted Class

In the previous section, we derive a minimax solution to (2) under the assumption that $p_{\theta }\in P\left(\Theta_{r}\right)$ (equivalently, $\theta \in \Theta_{r}$), with high confidence. Unfortunately, it does not provide any guarantee for the event where $p_{\theta }\notin P\left(\Theta_{r}\right)$. We now consider a general setup where $p_{\theta }$ is not necessarily in $P(\Theta_{r})$ as well as introduce a more robust approach which addresses this case.

Let P(Θ) be a (non-restricted) model class that is known to contain the true parametric model $p_{\theta }$. Here, we define Θ as the set of all possible parameter values, such that θ∈Θ. For example, P(Θ) is a class of all normal distributions with an unknown mean and a unit variance (Θ=R), in the normal mean example above. As before, we would like to find a distribution *q* that minimizes $D_{\mathrm{KL}}(p_{\theta }\left||q\right)$. Simple calculus shows that

(4) $$\begin{array}{c} D_{\mathrm{KL}}(p_{\theta }\left||q\right)=D_{\mathrm{KL}}(p_{\theta }\left|\right|p_{\theta^{\prime }})+\int p_{\theta }\left(x\right)\log\frac{p_{\theta^{\prime }}\left(x\right)}{q(x)}dx \end{array}$$

for any choice of $p_{\theta^{\prime }}$. Specifically, (4) holds for any model in the restricted model class, $p_{\theta^{\prime }}\in P\left(\Theta_{r}\right)$. Notice that in this case, the first term of (4) is an error induced by the restrictive model class, independent of the choice of *q*. The second term is the residual, which depends on *q*. Notice that the first term only depends on $p_{\theta }$ and the reference distribution $p_{\theta^{\prime }}\in P\left(\Theta_{r}\right)$. This means that by choosing a model class $P(\Theta_{r})$ that is too “far” (or from the true distribution), we face a large overhead term that is independent of the estimator *q*. On the other hand, the second term depends on *q*, and may be universally bounded. In other words, we are interested in a universal bound of the form

(5) $$\begin{array}{cc} \; & \min_{q}\underset{p_{\theta }\in P\left(\Theta \right)}{\sup}\underset{p_{\theta^{\prime }}\in P\left(\Theta_{r}\right)}{\sup}\left(\int p_{\theta }\left(x\right)\log\frac{p_{\theta^{\prime }}\left(x\right)}{q(x)}dx\right). \end{array}$$

Interestingly, notice that (5) may be equivalently written as

(6) $$\begin{array}{cc} \; & \min_{q}\underset{p_{\theta }\in P\left(\Theta \right)}{\sup}\underset{p_{\theta^{\prime }}\in P\left(\Theta_{r}\right)}{\sup}\left(\int p_{\theta }\left(x\right)\log\frac{p_{\theta }\left(x\right)}{q(x)}dx-\int p_{\theta }\left(x\right)\log\frac{p_{\theta }\left(x\right)}{p_{\theta^{\prime }}\left(x\right)}dx\right)=\\ \; & \min_{q}\underset{p_{\theta }\in P\left(\Theta \right)}{\sup}\left(D_{\mathrm{KL}}(p_{\theta }\left||q\right)-\underset{p_{\theta^{\prime }}\in P\left(\Theta_{r}\right)}{\inf}D_{\mathrm{KL}}(p_{\theta }\left|\right|p_{\theta^{\prime }})\right). \end{array}$$

This means that (5) is just a constrained variant of (2). Therefore, similarly to (2), we would like to represent (5) as a (constrained) channel capacity problem.

**Definition** **1.** *Let $p_{\theta }\in P\left(\Theta \right)$ be an unknown probability distribution. Let $P(\Theta_{r})$ be a restrictive model class (that does not necessarily contain $p_{\theta }$). Assume that $\Theta_{r}$ is bounded. Define*

(7) $$\begin{array}{cc} F(\Theta ,\Theta_{r})≜ & \underset{\pi (\theta )}{\sup}\int_{\theta \in \Theta }\pi \left(\theta \right)\left(D_{\mathrm{KL}}(p_{\theta }\left|\right|q_{\pi })-\min_{\theta^{\prime }\in \Theta_{r}}D_{\mathrm{KL}}(p_{\theta }\left|\right|p_{\theta^{\prime }})\right)d\theta =\\ \; & \underset{\pi (\theta )}{\sup}\left(I\left(T;X\right)-E_{\pi (\theta )}\min_{\theta^{\prime }\in \Theta_{r}}D_{\mathrm{KL}}(p_{\theta }\left|\right|p_{\theta^{\prime }})\right). \end{array}$$

As in (2), I(T;X) is the mutual information between a source variable T∼π, and a target variable *X*, that is characterized by the transition probabilities P(Θ). The constraint is simply the expected divergence (with respect to π) between $p_{\theta }$ and its closest projection in $\Theta_{r}$. The term $q_{\pi }$ is a mixture distribution over P(Θ), according to the prior π. Notice that here, the mixture is over the non-restricted model class, as opposed to (2), where the mixture is over $P(\Theta_{r})$. We denote this distribution, $q_{\pi }$, as the projected mixture distribution.

**Theorem** **2.** *Let $p_{\theta }\in P\left(\Theta \right)$ be an unknown probability distribution. Let $P(\Theta_{r})$ be a restrictive model class (that does not necessarily contain $p_{\theta }$). Assume that $\Theta_{r}$ is bounded. Then, for $F(\Theta ,\Theta_{r})\infty$ the following holds:*

(8) $$\begin{array}{c} \min_{q}\underset{p_{\theta }\in P\left(\Theta \right)}{\sup}\underset{p_{\theta^{\prime }}\in P\left(\Theta_{r}\right)}{\sup}\left(\int p_{\theta }\left(x\right)\log\frac{p_{\theta^{\prime }}\left(x\right)}{q(x)}dx\right)=F\left(\Theta ,\Theta_{r}\right). \end{array}$$

A proof of Theorem 2 is provided in Appendix A. Theorem 2 establishes a redundancy-capacity result, similarly to (2). It shows that (5) may be obtained by solving a constrained channel capacity problem, and the distribution which achieves it is, again, a mixture distribution. This result is further discussed in [28] in a different (asymptotic) setup.

In addition, notice that for a bounded $\Theta_{r}$ the formulation in (5) may be equivalently written as

$$\min_{q}\underset{p_{\theta }\in P\left(\Theta \right)}{\sup}\int p_{\theta }\left(x\right)\log\frac{p_{\theta }^{*}\left(x\right)}{q(x)}dx,$$

where $p_{\theta }^{*}={\mathrm{argmin}}_{p_{\theta^{\prime }}\in P\left(\Theta_{r}\right)}D_{\mathrm{KL}}(p_{\theta }\left|\right|p_{\theta^{\prime }})$. In other words, $F(\Theta ,\Theta_{r})$ is also the optimal universal minimizer of the second term of (4), for a specific (greedy) choice of $p_{\theta^{\prime }}\in P\left(\Theta_{r}\right)$ that minimizes the first term. This result may be viewed as a “triangle inequality” for the KL divergence: given a reference set $P(\Theta_{r})$, the KL divergence $D_{\mathrm{KL}}(p_{\theta }\left||q\right)$ is bounded from above by the closest projection in $P(\Theta_{r})$ to $p_{\theta }$, plus an overhead-term $F(\Theta ,\Theta_{r})$. It is important to emphasize that $F(\Theta ,\Theta_{r})$ is not new to the universal coding literature. In fact, it was introduced in [17] as relative redundancy, in the context of robust codes for universal compression. However, it was mostly studied in an asymptotic regime, where *n* i.i.d. variables $X^{n}$ are simultaneously compressed. However, it was mostly studied in an asymptotic regime, where *n* i.i.d. variables *X^n^* are simultaneously compressed [28].

Similarly to the channel capacity problem, the term $F(\Theta ,\Theta_{r})$ holds a closed-form analytical expression only in several special cases. Therefore, we introduce a simple iterative algorithm, which provides an optimal solution to it (as indicated in [28]). Our suggested routine is similar in spirit to the Blahut–Arimoto algorithm [21], which is typically applied to intractable channel capacity problems.

**Theorem** **3.** *Let P(Θ) and $P(\Theta_{r})$ be two model classes. Let $F(\Theta ,\Theta_{r})$ follow the definition above. Assume that $\Theta_{r}$ is bounded. Then, for $F(\Theta ,\Theta_{r})\infty$ the following holds:*

(9) $$\begin{array}{c} F\left(\Theta ,\Theta_{r}\right)=\underset{\phi (\theta ),\psi (\theta ,x)}{\sup}\int_{\theta \in \Theta }\int_{x}\phi \left(\theta \right)p_{\theta }\left(x\right)\log\frac{\psi (\theta ,x)}{\phi (\theta )}\frac{p_{\theta }^{*}\left(x\right)}{p_{\theta }\left(x\right)}dxd\theta \end{array}$$

*where ϕ(θ) and ψ(θ,x) are probability distributions (over the variable θ, for any given x), and $p_{\theta }^{*}\left(x\right)={\mathrm{argmin}}_{\theta^{\prime }\in \Theta_{r}}D_{\mathrm{KL}}(p_{\theta }\left|\right|p_{\theta^{\prime }})$. Further, the solution to (9) may be attained by the following iterative projection algorithm:*

1. *For a fixed ϕ(θ), we set $\psi \left(\theta ,x\right)=\frac{\phi \left(\theta \right)p_{\theta }\left(x\right)}{\int_{\theta \in \Theta }\phi \left(\theta \right)p_{\theta }\left(x\right)d\theta }$*
2. *For a fixed ψ(θ,x), we set $\phi \left(\theta \right)=\frac{\prod_{x}{\tilde{\psi }\left(\theta ,x\right)}^{p_{\theta }\left(x\right)}}{\int_{\theta \in \Theta }\prod_{x}{\tilde{\psi }\left(\theta ,x\right)}^{p_{\theta }\left(x\right)}d\theta }$ where $\tilde{\psi }\left(\theta ,x\right)=\psi \left(\theta ,x\right)\frac{p_{\theta }^{*}\left(x\right)}{p_{\theta }\left(x\right)}$.*

*Finally, the distribution q that achieves $F(\Theta ,\Theta_{r})$ is given by $q_{\Theta }=\int_{\theta \in \Theta }\phi^{*}\left(\theta \right)p_{\theta }d\theta$, where $\phi^{*}\left(\theta \right)$ is ϕ(θ) at the final iteration of the algorithm.*

A proof for this theorem is provided in Appendix B.

In many practical cases, the choice of a model class P(Θ) is not a trivial task. For example, consider a real-world setup where a domain expert suggests that the underlying model follows a Normal distribution with an unknown mean. However, we would like to design a scheme that does not heavily rely on this assumption. Therefore, we may consider the general case where P(Θ) is the simplex of all possible probability distributions. This important special case described in the following section.

### The Normalized Maximum Likelihood

Consider a model class P(Θ)=P={p|p(x)≥0,∫p(x)dx=1}. Here, the solution to (7) holds a closed form expression.

**Theorem** **4.** *Let $p_{\theta }\in P$ be an unknown probability distribution where P={p|p(x)≥0,∫p(x)dx=1}. Let $P(\Theta_{r})$ be a restrictive model class. Assume that $\Theta_{r}$ is bounded and $Z≜\int \max_{p_{\theta^{\prime }}\in P\left(\Theta_{r}\right)}p_{\theta^{\prime }}\left(x\right)dx$. Let $\Gamma \left(\Theta_{r}\right)≜\log\left(Z\right)$. For $\Gamma (\Theta_{r})\infty$,*

$$\min_{q}\underset{p_{\theta }\in P}{\sup}\underset{p_{\theta^{\prime }}\in P\left(\Theta_{r}\right)}{\sup}\int p_{\theta }\left(x\right)\log\frac{p_{\theta^{\prime }}\left(x\right)}{q(x)}dx≜\Gamma \left(\Theta_{r}\right)$$

*and the model q that achieves the minimum is the normalized maximum likelihood (NML) [29], $q_{nml}\left(x\right)=\max_{p_{\theta^{\prime }}\in P\left(\Theta_{r}\right)}p_{\theta^{\prime }}\left(x\right)/Z$*

This theorem is an immediate application of Shtarkov’s NML result [29]. It suggests that given a model class $P(\Theta_{r})$ which does not necessarily contain the true model *p*, the NML estimator $q_{nml}$ minimizes the worst-case regret over all possible distributions and guarantees an overhead of at most $\Gamma (\Theta_{r})$ bits, compared to the best model in the class $P(\Theta_{r})$, for any possible *p*. Further, it is shown that this result is tight, in the sense that there exist probability distributions p∈P(Θ) and $p_{\theta^{\prime }}\in P\left(\Theta_{r}\right)$ that achieve the $\Gamma (\Theta_{r})$ term. Notice that for every P(Θ) and $P(\Theta_{r})$ that satisfy the conditions above, we have that $C\left(\Theta_{r}\right)\leq F\left(\Theta ,\Theta_{r}\right)\leq \Gamma \left(\Theta_{r}\right).$ This means that under more restrictive assumptions we attain tighter worst-case performance guarantees, as expected. We now demonstrate our suggested methods in synthetic and real-world problems.

## 5. The Normal Distribution

Let us first study the classical unknown mean problem in the Gaussian case. Consider *n* i.i.d. samples, drawn from a *d*-dimensional multivariate normal distribution with an unknown mean μ and a known covariance matrix Σ. The 100(1−α)% confidence region for μ is $M_{r}=\left\{\mu |{\left(\bar{x}-\mu \right)}^{T}\Sigma^{-1}\left(\bar{x}-\mu \right)\leq \frac{1}{n}\chi_{d}^{2}\left(1-\alpha \right)\right\}$, where $\bar{x}$ is the sample mean, and $\chi_{d}^{2}$ is a Chi-squared distribution with *d* degrees of freedom. First, we would like to solve the minimax problem (2) with respect to $M_{r}$. We apply the redundancy-capacity theorem (3) and define a corresponding channel, X=M+Z, where *M* is a random vector, taking values over the domain $M_{r}$, while Z∼N(0,Σ), independent of *M* (see [30] for detail). This formulation is also known as an amplitude-constrained capacity problem. We show (Appendix C) that it is equivalent to the generic case where $Z∼N(0,I_{d})$ and $M\in M_{r}^{\prime }$ where $M_{r}^{\prime }=\left\{\mu |\mu^{T}\mu \leq \frac{1}{n}\chi_{d}^{2}\left(1-\alpha \right)\right\}$. Notice that the domain of *M* is now restricted to a *d*-dimensional ball (defined by $M_{r}^{\prime }$) and our goal is to find the capacity achieving distribution of *M*. It has been shown [31] that the solution to this problem is achieved when *M* is supported on a finite number of concentric spheres. Recently, the authors of [32] studied the necessary conditions under which the solution is a single sphere, centered at the origin. Specifically, they derived the largest radius $r_{d}$ for which the capacity achieving distribution is uniform on the sphere of the *d*-dimensional ball. This means that if the radius defined by $M_{r}^{\prime }$ is smaller than $r_{d}$, then the solution to our minimax problem is immediate. Applying Dytso et al. analysis to our problem, we attain the following result.

**Theorem** **5.** *Let $x^{n}$ be a collection of n i.i.d. samples from a d-dimensional multivariate normal distribution with an unknown mean μ and a known covariance matrix Σ. Let $M_{r}$ be a 100(1−α)% confidence region for μ. Let $r_{d}$ be the largest radius for which the capacity achieving distribution is uniform on the sphere of a d-ball, as defined in Table 1 of [32]. Then, for any $n\geq \chi_{d}^{2}\left(1-\alpha \right)/r_{d}^{2}$, the solution to the minimax problem (2) over the confidence region $M_{r}$ is attained by a uniform mixture of Gaussians with means on the confidence region, $q_{\pi }\propto \int_{\mu \in O(M_{r})}N\left(\mu ,\Sigma \right)d\mu$ where $O\left(M_{r}\right)=\left\{\mu |{\left(\bar{x}-\mu \right)}^{T}\Sigma^{-1}\left(\bar{x}-\mu \right)=\frac{1}{n}\chi_{d}^{2}\left(1-\alpha \right)\right\}$.*

**Figure 1:**
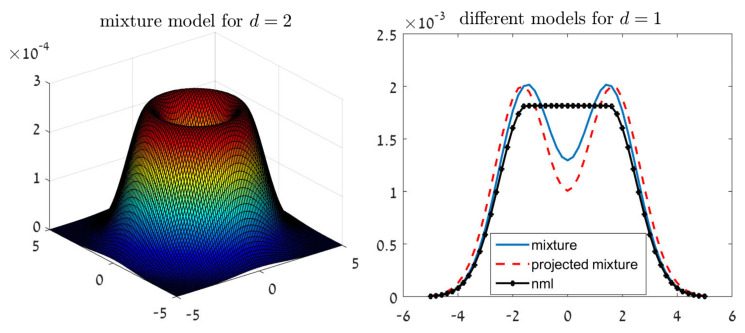
The shape of our suggested solutions in the unknown normal mean problem. (**Left**)—the mixture distribution $q_{\pi }$ for d=2. (**Right**)—all methods for d=1 and an example confidence interval of [−1.5,1.5].

For example, let α=0.05 and d=2. We have that $r_{d}=2.454$ (as appears in Table 1 of [32]), and the solution to (2) over $M_{r}$ is given by $q_{\pi }\propto \int_{\mu \in O(M)}N\left(\mu ,\Sigma \right)d\mu$, for every n≥1. The left chart of Figure 1 illustrates the shape of $q_{\pi }$ in this case. This Gaussian mixture shape may seem counterintuitive at a first glance, as $x^{n}$ are known to be drawn from a normal distribution. However, the reason is quite clear. Our inference criterion strives to control a set of Gaussian models. Therefore, the optimal solution is not necessarily the most likely model in the set, but a mixture of models.

Let us now turn to the projected mixture distribution and the NML. The right chart of Figure 1 demonstrates the shape of these estimators for d=1 and $M_{r}=\left[-1.5,1.5\right]$.

First, we notice that the projected mixture is again a Gaussian mixture, with means outside of the confidence interval. On the other hand, the NML solution is not a Gaussian mixture; simple calculus shows that

(10) $$\begin{array}{c} q_{nml}\left(x\right)\propto \left\{\begin{array}{cc} \frac{1}{\sqrt{2\pi }}\exp\left(-\frac{1}{2}{\left(x+a\right)}^{2}\right) & x-a\\ \frac{1}{\sqrt{2\pi }} & -a\leq x\leq a\\ \frac{1}{\sqrt{2\pi }}\exp\left(-\frac{1}{2}{\left(x-a\right)}^{2}\right) & xa \end{array}\right. \end{array}$$

for a symmetric confidence interval [−a,a].

**Figure 2:**
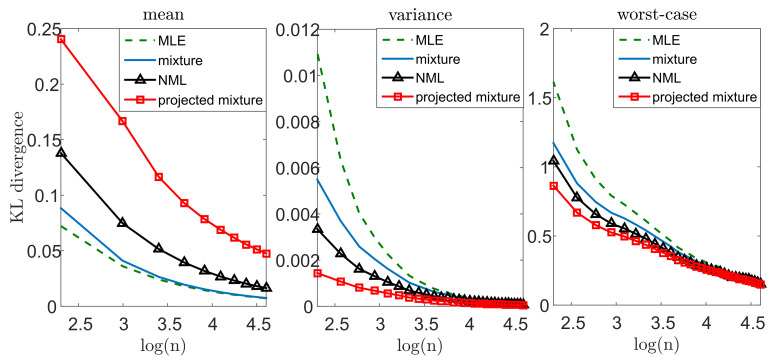
Mean, variance, and worst-case performance in the Gaussian unknown mean problem. We draw *n* samples from $p_{\mu }∼N\left(0,1\right)$ and compute $D_{\mathrm{KL}}(p_{\mu }||q\left(\cdot |x^{n}\right))$. We repeat this experiment 10,000 times and evaluate the mean (**left**), variance (**middle**), and worst-case (**right**) performance.

Let us illustrate the performance of our suggested methods. We draw *n* i.i.d. samples from a standard normal distribution $p_{\mu }∼N\left(0,1\right)$ where the mean μ=0 is unknown and the variance is known. We apply our suggested methods (with α=0.05) and evaluate the KL divergence from the true distribution, $D_{\mathrm{KL}}(p_{\mu }||q\left(\cdot |x^{n}\right))$. We compare our results with the performance of the MLE, $D_{\mathrm{KL}}\left(p|\right|q_{\mathrm{mle}}\left(x^{n}\right))$, where $q_{\mathrm{mle}}\left(x^{n}\right)=N\left(\bar{x},1\right)$. Notice that the MLE is also known to be the minimax solution to (1) in this setting. We repeat this experiment *k* = 10,000 times, for different sample sizes *n*. For each *n* we evaluate the mean $E_{x^{n}∼p_{\mu }^{n}}D_{\mathrm{KL}}(p_{\mu }||q\left(\cdot |x^{n}\right))$, the variance ${\mathrm{var}}_{x^{n}∼p_{\mu }^{n}}D_{\mathrm{KL}}(p_{\mu }||q\left(\cdot |x^{n}\right))$ and the worst-case $\max_{x^{n}\in X_{k}}D_{\mathrm{KL}}(p_{\mu }||q\left(\cdot |x^{n}\right))$, where $X_{k}$ is the set of *k* random draws of $x^{n}$ from $p_{\mu }^{n}$. Figure 2 demonstrates our results. Notice that we lose some accuracy, on the average, with all of our methods, compared to the MLE. On the other hand, the variance of the MLE is significantly greater, which suggests that it is less reliable for a given instance of data. Finally, we notice a significant gain in the worst-case performance. This behavior is not surprising: our approach strives to control the worst-case performance for each given draw. In this sense, we may view our approach as an “insurance policy”—we pay a small cost on the average, but attain a more stable estimator and gain significantly if “something bad happens” (that is, we observe $x^{n}$ that do not represent the true model well enough). Notice that this phenomenon is more evident when the inference problem is more challenging (smaller *n*’s). As we compare our suggested models to each other, we notice that the mixture distribution is the most conservative (that is, smallest cost and smallest gain), while the projected mixture is the least conservative. The reason is quite clear: in about (1−α) of the draws, the true parameter lies within the confidence region, and the mixture distribution is closer to it. This implies better performance on the average and worse performance in the extremes. Interestingly, the NML behaves as a compromise between the two. This is mostly as the NML does not assume that $\mu \in M_{r}$ (better than the mixture in the worst-case). However, it also unnecessarily controls non-Gaussian models (worse than the projected mixture).

**Figure 3:**
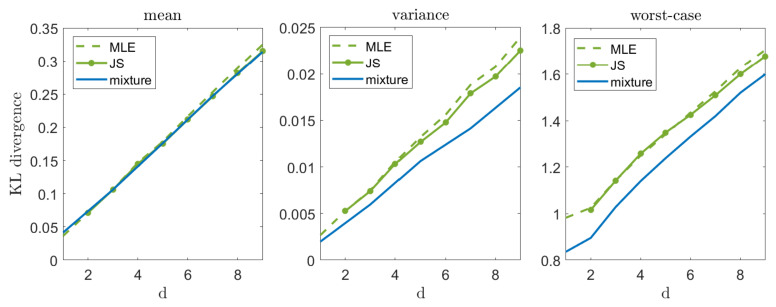
Mean, variance, and worst-case performance in high-d Gaussian unknown mean problem. In each experiment we draw n=20 samples from $p=N(\underline{1},I_{d})$ and compute $D_{\mathrm{KL}}(p||q\left(\cdot |x^{n}\right))$. We repeat this experiment 10,000 times and evaluate the mean (**left**), variance (**middle**), and worst-case (**right**) performance.

Let us now illustrate our suggested approach in a high-dimensional setting, $p=N(\underline{1},I_{d})$. Figure 3 compares the mixture estimator (which demonstrates a reasonable compromise between mean and worst-case performance) with the MLE and the James–Stein (JS) estimator. As we can see, the JS estimator slightly outperforms MLE on the average (as discussed in [3]), while the mixture distribution is very close to them. However, as we focus on the variance and the worst-case performance, the mixture distribution demonstrates a significant improvement, as expected. It is important to mention that in a zero mean case, the JS estimator achieves a significantly lower mean error (as discussed in [2]) and a remarkable increase in variance and worst-case performance. These results are omitted for brevity.

## 6. The Multinomial Distribution

We now turn to an additional important example of finite alphabet distributions. Let $x^{n}$ be *n* i.i.d. draws from a multinomial distribution over an alphabet size *m*. Notice that here, the parametric family spans the entire simplex. Therefore, we omit the parametric subscript θ to avoid an overload of notation, and regard *p* as the unknown vector of parameters. As discussed above, we would first like to construct a minimal-volume confidence region for *p*, denoted as $P_{r}$. Unfortunately, there exists no closed-form solution in the multinomial case. Therefore, we turn to an approximate confidence region suggested in [33]. As many other approximation techniques [34,35], Sison and Glaz derive a rectangular region $P_{sg}=\left\{p|\;p_{l}\left(i\right)\leq p\left(i\right)\leq p_{u}\left(i\right)\;\forall i=1,\cdots ,m\right\}$ which demonstrates a smaller expected volume compared to alternatives. Our first step is to define a subset of Sison and Glaz region, $P_{r}\subset P_{sg}$, which corresponds to valid probability distributions, $P_{r}=\left\{p|p\in P_{sg},\;\sum p\left(i\right)=1\right\}$. Notice that $P_{r}$ is a convex set, and denote its set of vertexes as $V(P_{r})$. We show (Appendix D) that the solution to (2) over $P_{r}$ is attained by solving (3) over $V(P_{r})$. This means that instead of considering the entire class $P_{r}$, we only need to focus on the discrete set $V(P_{r})$.

Unfortunately, there exists no closed-form solution to (3) in this setting. However, as the cardinality of π is finite (as we optimize over $V(P_{r})$), we may apply the Blahut–Arimoto algorithm [21] and attain a numerical solution, at a relatively small computational cost. Finally, we derive the projected mixture and the NML. As mentioned above, the parametric family spans the entire simplex. This means that the two methods are identical, and obtained by applying the NML over $P_{r}$.

We now demonstrate our suggested approach. Let $x^{n}$ be i.i.d. draws from a Zipf’s law distribution over an alphabet size m=5 and a parameter s=1.01, $p\left(i\right)\propto i^{-s}$. The Zipf’s law distribution is a commonly used benchmark distribution, mostly in modeling of natural (real-world) quantities. It is widely used in physical and social sciences, linguistics, economics, and many other fields [36,37,38]. As in Section 5, we compare our suggested methods to the MLE. In addition, we consider the popular Laplace estimator, which adds a single count to all events, followed by a MLE. In our experiments we focus on an enhanced variant of Laplace [39], which adds 1/2 to all events, $q_{\mathrm{lap}}\left(n_{i}\right)\propto n_{i}+1/2$, where $n_{i}$ is the number of appearances of the i^th^ symbol in $x^{n}$. This variant holds important universality properties and is widely known as the Krichevsky–Trofimov estimator [39,40].

**Figure 4:**
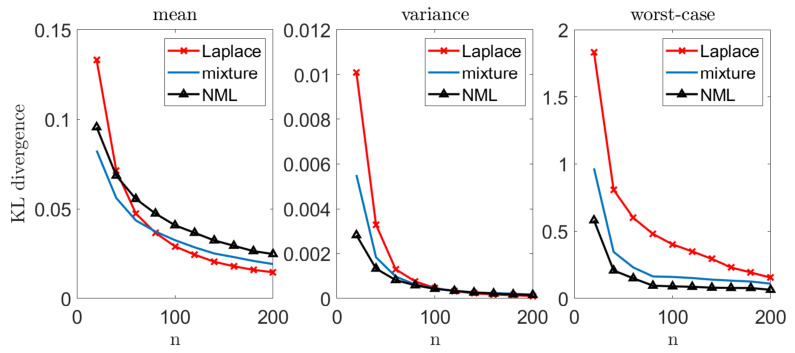
Multinomial inference. In each experiment, we draw *n* samples from a Zipf’s law distribution with m=5 and s=1.01. We evaluate $D_{\mathrm{KL}}(p||q\left(\cdot |x^{n}\right))$ for different estimators. We repeat this experiment 10,000 times and report the mean (**left**), variance (**middle**), and worst-case (**right**) performance.

We repeat each experiment *k* = 10,000 times and report the estimated mean, variance, and worst-case performance, as in the Gaussian case. Figure 4 demonstrates the results we achieve. We omit the MLE as it typically results in an unbounded divergence (in cases where at least a single symbol fails to appear).

As in the unknown normal mean problem, we notice that in more challenging setups (small *n*), our worst-case gain is quite remarkable. This gain narrows down as *n* increases, and all the estimators converge to the same solution. In addition, we observe a significant gain in expectation when *n* is small. It is important to emphasize that when the underlying distribution is easier to infer (all p(x) are bounded away from zero, as with the uniform distribution), the advantage of using the minimax approach is less evident (similarly to the large *n* regime in the Zipf’s law example).

## 7. Large Alphabet Probability Estimation

In the large alphabet regime, we study a multinomial distribution where mn. This problem has been extensively studied over the years, with many applications ranging from language processing to biological studies [41]. Here, traditional methods like MLE are typically ineffective, as they assign a zero probability to unseen events. Several alternatives have been suggested over the years. In his seminal work, Laplace [42] suggested to add a single count to all events, followed by a maximum likelihood estimator. The work of Laplace was later generalized to a class of add-constant estimators [39], with the important special case of the Krichevsky–Trofimov estimator (as discussed in Section 6). A significant milestone in the history of large alphabet probability estimation was established in the work of Good and Turing [43]. Their approach suggests that unseen events shall be assigned a probability proportional to the number of events that appear once. To this day, Good–Turing estimators are the most commonly used methods in practical problems (see, for example, Section 1.4 in [41]). Despite the great interest in large alphabet estimation, provably-optimal schemes remain elusive [41]. Moreover, the accuracy of existing methods do not allow us to construct practical confidence regions. In fact, Paninski [44] showed that in the large alphabet setup, the minimal expected worst-case divergence is unbounded, and grows like log(m/n). Therefore, it is quite difficult to define a small enough restrictive model class that contains *p* with high confidence. In this case, we introduce an alternative approach for the design of $P_{r}$, followed by an NML estimator.

### The Leave-One-Out Hypothesis Class

Define the convergence rate of $q(\cdot |x^{n})$ as $\Delta_{p}\left(n\right)=$$E\left(D_{\mathrm{KL}}(p||q\left(\cdot |x^{n}\right))-D_{\mathrm{KL}}(p||q\left(\cdot |x^{n+1}\right))\right).$ We say that an estimator $q(\cdot ;x^{n})$ is *proper* if it satisfies, for every *p*,

- $ED_{\mathrm{KL}}(p||q\left(\cdot |x^{n}\right))\infty$ for all n≥0
- $\Delta_{p}\left(n\right)\geq 0$ for all $n\geq n_{0}$
- $\Delta_{p}\left(n\right)$ is monotonically non-increasing for all $n\geq n_{0}$

The first condition states that the expected loss is finite for any *n*. The second condition indicates that asymptotically, adding more samples only improves the expected accuracy. The third condition says that the rate of the improvement is non-increasing in the number of samples. For example, the improvement from 100 to 101 samples is greater than the improvement from 1000 to 1001 samples, on the average. We now define the *leave-one-out* model class. Let $x_{\left[-i\right]}^{n-1}=\left\{x_{1},...,x_{i-1},x_{i+1},...,x_{n}\right\}$ be the leave-one-out set of $x^{n}$, excluding the $i^{th}$ sample. Let $q(\cdot |x_{\left[-i\right]}^{n-1})$ be the corresponding proper estimate. The leave-one-out (loo) model class is defined as $P_{loo}={\left\{q\left(\cdot |x_{\left[-i\right]}^{n-1}\right)\right\}}_{i=1}^{n}$. Theorem 6 below establishes that on the average, the accuracy of the best model in $P_{loo}$ is bounded from above by accuracy of $q(\cdot |x^{n})$, plus an additional vanishing overhead term.

**Theorem** **6.** *Let q be a proper estimator. Then,*

(11) $$\begin{array}{c} E\left(\min_{i}D_{\mathrm{KL}}\left(p||q\left(\cdot |x_{\left[-i\right]}^{n-1}\right)\right)\right)\leq E\left(D_{\mathrm{KL}}\left(p||q\left(\cdot |x^{n}\right)\right)\right)+o\left(\frac{1}{n}\right). \end{array}$$

A proof for this Theorem is provided in Appendix E. Notice that the inequality is due to the convexity of the different operators. This means that typically, we expect the inequality to be strict. In other words, given a proper estimator *q*, Theorem 6 shows that on the average, there exists at least a single model in $P_{loo}$ that is better than $q(\cdot |x^{n})$, up to a vanishing overhead term of $o\left(\frac{1}{n}\right)$. In Appendix F we show that any add-constant (Laplace) estimator satisfies (11). Further, our experiments indicate that the same property holds for the Good–Turing estimator. This motivates the use of these estimators in the design of $P_{r}=P_{loo}$, as suggested by (4).

**Figure 5:**
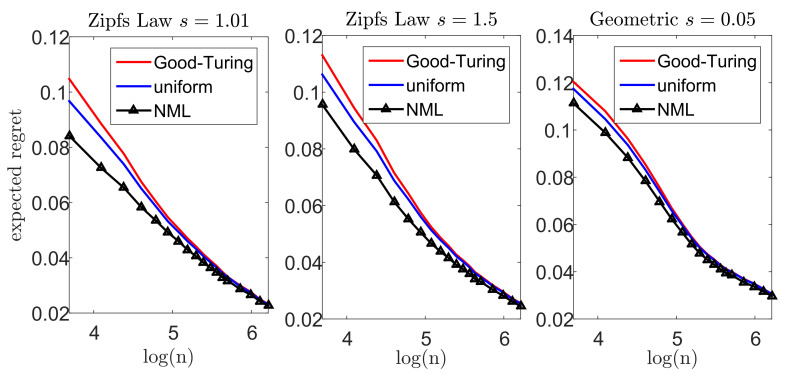
Large alphabet probability estimation- the expected regret (difference) between $D_{\mathrm{KL}}(p||q\left(\cdot |x^{n}\right))$ and the performance of the natural oracle, $D_{\mathrm{KL}}\left(p|\right|p_{nat}\left(\cdot |x^{n}\right))$.

**Figure 6:**
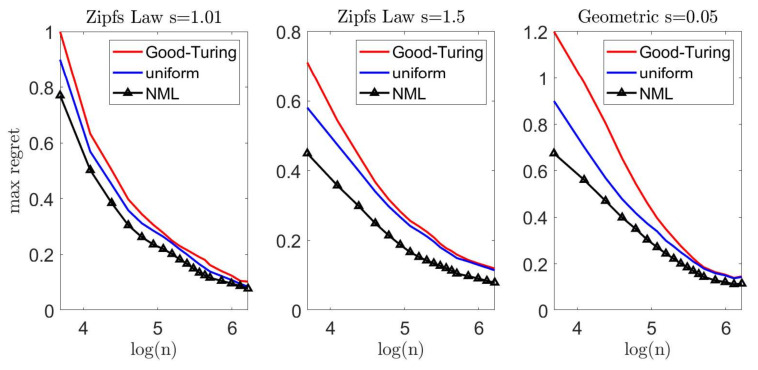
Large alphabet probability estimation—the worst-case regret between $D_{\mathrm{KL}}(p||q\left(\cdot |x^{n}\right))$ and the performance of the natural oracle, $D_{\mathrm{KL}}\left(p|\right|p_{nat}\left(\cdot |x^{n}\right))$.

Let us now demonstrate our suggested scheme. In each experiment, we draw *n* samples from a multinomial distribution over an alphabet size m=1000. We apply the Krichevsky–Trofimov estimator, $q\left(n_{i}\right)\propto n_{i}+1/2$, and a Good–Turing estimator, following the implementation of Gale [45]. We compare these estimators to our suggested scheme; we construct a loo model class using Good–Turing, followed by an NML estimator. In addition, we compare the NML with a simple uniform average over the loo model class. A comprehensive description of our suggested scheme is provided in Appendix G. To emphasize the difference between the suggested schemes, we compare each estimator with a *natural oracle*$p_{nat}\left(x^{n}\right)$; an estimator who knows the true model *p*, but is restricted to assign the same probability to symbols that appear the same number of times in $x^{n}$. The performance of this oracle serves as a lower bound [41]. Figure 5 and Figure 6 demonstrate the results we achieve for a Zipf’s law distribution $p\left(i\right)\propto i^{-s}$ with a parameter value of s=1.01 (left) and s=1.5 (center). In addition, we consider a geometric distribution $p\left(i\right)={\left(1-s\right)}^{i-1}s$ with s=0.05 (right). We report the expected difference (regret) between $D_{\mathrm{KL}}(p||q\left(\cdot |x^{n}\right))$ and $D_{\mathrm{KL}}\left(p|\right|p_{nat}\left(\cdot |x^{n}\right))$ in Figure 5, while the worst-case regret is presented in Figure 6. We omit the uncompetitive performance of the Krichevsky–Trofimov estimator.

As we observe Figure 5, we notice that our suggested NML method outperforms Good–Turing when the alphabet size is relatively small. As *n* increases, the improvement becomes less evident as the restrictive model class converges to $q(\cdot |x^{n})$. Further, we notice that a uniform average over the loo model class is also favorable, but demonstrates a slighter improvement. Finally, we compare the worst-case performance in Figure 6. Here, again, we notice a significant improvement as in the previous experiments. For example, for n=40 and a Zipf’s law distribution (s=1.5), the Good–Turing results in a regret of 0.72 bits while the uniform mixture is 0.58 bits and the NML is only 0.43 bits.

## 8. Real-World Example

Let us now introduce a real-world example. The Wisconsin breast cancer study (https://archive.ics.uci.edu/ml/datasets/Breast+Cancer+Wisconsin+(Diagnostic), accesed on 14 June 2021) considers 569 diagnosed tumors, of which 357 are benign (B) and 212 are malignant (M) [46]. Each tumor is characterized by 32 features, including its size, texture, surface, and more. We would like to study the radius of benign tumors and assess its probability function. This probability is of high interest as it allows us, for example, to control type-I error in a future hypothesis testing (the probability of deciding a tumor is malignant, given that it is benign).

The medical domain knowledge suggests that the size of the tumor follows a normal distribution, with different parameters for the B and M tumors. Therefore, the standard approach is to estimate the parameters from the given data. For simplicity, we assume the true variance is known (estimated from the entire population) and focus on the unknown mean.

**Figure 7:**
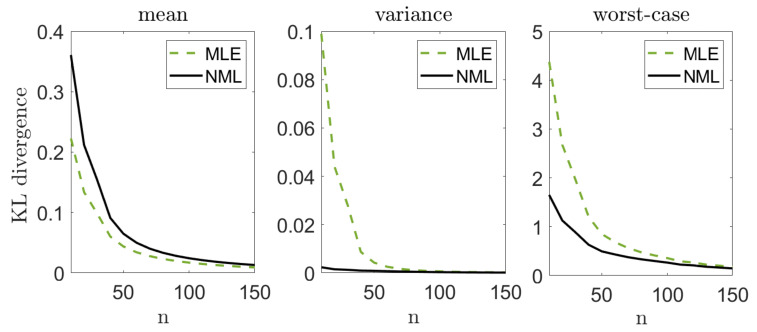
Breast cancer tumor study. Mean, variance, and worst-case performance of different estimators, based on *n* samples.

As in the previous sections, we study the performance of different estimation schemes. We draw *n* samples from the B class, and apply the MLE and the suggested NML scheme. Notice that we focus on the NML as it is the most robust approach for the modeling assumption (and henceforth most suitable for such a clinical trail). We repeat this experiment 10,000 times for every value of *n* and report the mean, variance, and worst-case KL divergence between the “true empirical distribution” (based on all the B samples that we have) and each estimator that we examine. Figure 7 demonstrates the results we achieve. As we can see, our suggested approach attains a significantly better worst-case results.

It is important to emphasize that the MLE is the solution to the classical minimax estimation scheme (1), under the assumption that the data is generated from normal distribution (see Section 2). Our approach with the NML relaxes this strong restriction and attains a significant improvement in the worst-case performance.

## 9. Discussion and Conclusions

In this work, we study a minimax inference framework. Our suggested scheme considers a class of models, defined by the parametric confidence region of the given samples. Then, we control the worst-case performance within this class. Our formulation relaxes some strong modeling assumptions of the classical minimax framework and considers a robust inference scheme for the complete unknown distribution. The attained solution draws fundamental connections to basic concepts in information theory. We demonstrate the performance of our suggested framework in classical inference problems, including normal and multinomial distributions. In addition, we demonstrate our suggested scheme on more challenging large alphabet probability estimation problems. Finally, we study a real-world breast cancer example. In all of these settings we introduce a significant improvement in the worst-case, at a typically low cost on the average. This demonstrates an “insurance-like” trade-off; we pay a small cost on the average to avoid a great loss if “something bad happens” (that is, the observed samples do not represent the true model well enough).

It is important to emphasize that our suggested scheme is not limited to confidence region model classes. In fact, in many cases, exact confidence regions are difficult to attain, or result in model classes that are too large to control (for example, large alphabet problems with many unseen symbols). In these cases, we consider alternative forms of “reasonable” classes of models. One possible solution is the leave-one-out (LOO) class, discussed in Section 7. Additional alternatives are bootstrap confidence regions, Markov Chain Monte Carlo (MCMC) sampling and others.

Finally, our suggested scheme may be generalized to a supervised learning framework. For example, consider a linear regression problem. The standard approach is to estimate the regression coefficients that best explain the data (typically by least squares analysis). However, notice we may also construct confidence intervals for the sought coefficients. This way, we can define a restricted model class (similarly to Section 3), and seek minimax estimates with respect to it. This idea may be generalized to more complex learning schemes such as deep neural networks. Specifically, we may construct a restricted model class as the vicinity of some class of parameters that the network converges to, and control the corresponding worst-case performance. We consider this direction for our future work.

## Data Availability

Not applicable.

## Appendix A. A Proof of Theorem 2

Let $p_{\theta }\in P\left(\Theta \right)$ be an unknown probability distribution. Let $P(\Theta_{r})$ be a restrictive model class that corresponds to a restricted set of parameters $\Theta_{r}$. We would like to solve the minimax problem

(A1) $$\begin{array}{cc} \; & \min_{q}\underset{\theta^{\prime }\in \Theta_{r}}{\sup}\underset{\theta \in \Theta }{\sup}\int p_{\theta }\left(x\right)\log\frac{p_{\theta^{\prime }}\left(x\right)}{q(x)}dx=\min_{q}\underset{\theta \in \Theta }{\sup}(D_{\mathrm{KL}}(p_{\theta }\left||q\right)-f_{\Theta_{r}}\left(p_{\theta }\right)), \end{array}$$

where $f_{\Theta_{r}}\left(p_{\theta }\right)≜\inf_{\theta^{\prime }\in \Theta_{r}}D_{\mathrm{KL}}(p_{\theta }\left|\right|p_{\theta^{\prime }})$. Let us now define an equivalent problem to (A1),

(A2) $$\begin{array}{c} \min_{q}\underset{\pi (\theta )}{\sup}\int_{\theta \in \Theta }\pi \left(\theta \right)\left(D_{\mathrm{KL}}(p_{\theta }\left||q\right)-f_{\Theta_{r}}\left(p_{\theta }\right)\right)d\theta . \end{array}$$

where π(θ) is a weight function satisfying π(θ)≥0 and $\int_{\theta \in \Theta }\pi \left(\theta \right)d\theta =1$. Notice that the equivalence holds since for a fixed *q*, a weight function which puts all probability mass on the worst θ is a least favorable function. Let us now change the order of the minimum and supremum, similarly to the redundancy-capacity theorem (3).

Let $M_{\Theta }$ be the collection of all measures (on *X*) that can be obtained as mixtures of the $p_{\theta }$ measures. Let ${\bar{M}}_{\Theta }$ be the closure of $M_{\Theta }$. Define

(A3) $$\begin{array}{cc} \; & \psi \left(q,\pi \left(\theta \right)\right)=\int_{\theta \in \Theta }\pi \left(\theta \right)\left(D_{\mathrm{KL}}(p_{\theta }\left||q\right)-f_{\Theta_{r}}\left(p_{\theta }\right)\right)d\theta \\ \; & \bar{V}=\min_{q}\underset{\pi (\theta )}{\sup}\psi \left(q,\pi \left(\theta \right)\right)\\ \; & \underline{V}=\underset{\pi (\theta )}{\sup}\min_{q}\psi \left(q,\pi \left(\theta \right)\right)\\ \; & \tilde{V}=\underset{\pi (\theta )}{\sup}\min_{q\in {\bar{M}}_{\Theta }}\psi \left(q,\pi \left(\theta \right)\right). \end{array}$$

Notice that if $F(\Theta ,\Theta_{r})\infty$, then $\tilde{V}=F\left(\Theta ,\Theta_{r}\right)$. This holds as for every π, the mixture $q\in {\bar{M}}_{\Theta }$, which minimizes ψ(q,π(θ)) is $q_{\pi }$ (see, for example, [27]). Therefore, we would like to show that $\bar{V}=\tilde{V}$. Here, we follow the steps of [27] and Sion’s minimax theorem [47].

**Theorem** **A1** (**Sion’s Minimax Theorem** [47]). *Let U be a compact convex subset of a linear topological space and V be a convex subset of a linear topological space. If f(u,v) is a real-valued function on U×V with*

1. *f(u,·) is upper semi-continuous and quasi-concave on V for all u∈U*
2. *f(·,v) is lower semi-continuous and quasi-convex on U for all v∈V*

* then, $\min_{u\in U}\sup_{v\in V}f\left(u,v\right)=\sup_{v\in V}\min_{u\in U}f\left(u,v\right)$.*

Let us first assume that P(Θ) is *uniformally tight*. In other words, for every ϵ0 there exists a compact set K⊆X such that $p_{\theta }\left(K\right)1-\epsilon$ for all $p_{\theta }\in P\left(\Theta \right)$. Haussler showed that if P(Θ) is uniformally tight, then it is totally bounded, and thus ${\bar{M}}_{\Theta }$ is compact [27]. Therefore, for $\tilde{V}\infty$ we have that

(A4) $$\begin{array}{cc} \bar{V}= & \min_{q}\underset{\pi (\theta )}{\sup}\psi \left(q,\pi \left(\theta \right)\right)\overset{\left(a\right)}{\leq }\min_{q\in {\bar{M}}_{\Theta }}\underset{\pi (\theta )}{\sup}\psi \left(q,\pi \left(\theta \right)\right)\overset{\left(b\right)}{=}\\ \; & \underset{\pi (\theta )}{\sup}\min_{q\in {\bar{M}}_{\Theta }}\psi \left(q,\pi \left(\theta \right)\right)\overset{\left(c\right)}{=}\underset{\pi (\theta )}{\sup}\min_{q}\psi \left(q,\pi \left(\theta \right)\right)=\underline{V} \end{array}$$

where:

(a) follows from definition
(b) follows from Sion’s minimax theorem
(c) for every π(θ), the distribution *q* which minimizes ψ(q,π(θ)) is a mixture distribution [27]. Notice that $f_{\Theta_{r}}\left(p_{\theta }\right)$ does not depend on *q*.

This means that $\bar{V}\leq \tilde{V}=\underline{V}$. On the other hand, it is easy to verify that $\bar{V}\geq \underline{V}$ due to the max-min inequality [48]. This means that $\bar{V}=\tilde{V}$ as desired.

Let us now assume that P(Θ) that is not uniformally tight. Haussler showed that in this case, $\sup_{\pi (\theta )}\min_{q}\int_{\theta \in \Theta }\pi \left(\theta \right)D_{\mathrm{KL}}(p_{\theta }||q)d\theta =\infty$ (Lemma 4 in [27]). Therefore, given that $f_{\Theta_{r}}\left(p_{\theta }\right)\infty$ for all θ∈Θ (as $\Theta_{r}$ is bounded), we have that $\underline{V}=\infty$. However, this contradicts $\tilde{V}\infty$.

## Appendix B. A Proof of Theorem 3

Assume that $\Theta_{r}$ is bounded. Let $p_{\theta }^{*}\left(x\right)={\mathrm{argmin}}_{\theta^{\prime }\in \Theta_{r}}D_{\mathrm{KL}}(p_{\theta }\left|\right|p_{\theta^{\prime }})$. Then,

(A5) $$\begin{array}{cc} F(\Theta ,\Theta_{r})≜ & \underset{\pi (\theta )}{\sup}\int_{\theta \in \Theta }\pi \left(\theta \right)\left(D_{\mathrm{KL}}(p_{\theta }\left|\right|q_{\pi })-\underset{\theta^{\prime }\in \Theta_{r}}{\inf}D_{\mathrm{KL}}(p_{\theta }\left|\right|p_{\theta^{\prime }})\right)d\theta =\\ \; & \underset{\pi (\theta )}{\sup}\int_{\theta \in \Theta }\pi \left(\theta \right)\left(D_{\mathrm{KL}}(p_{\theta }\left|\right|q_{\pi })-D_{\mathrm{KL}}(p_{\theta }\left|\right|p_{\theta }^{*})\right)d\theta =\\ \; & \underset{\pi (\theta )}{\sup}\int_{\theta \in \Theta }\pi \left(\theta \right)\left(\int_{x}p_{\theta }\left(x\right)\log\frac{p_{\theta }^{*}\left(x\right)}{q_{\pi }\left(x\right)}\right)d\theta . \end{array}$$

Similarly to the channel capacity problem, this optimization does not hold a closed form solution in the general case. Therefore, we introduce an alternating projection algorithm, similar in spirit to the Blahut–Arimoto algorithm [49,50]. For this purpose, we apply the well-known alternating maximization theorem (Lemma 9.4 and 9.5 in [51]).

**Lemma** **A1** **(The** **Alternating** **Maximization** **Theorem** **[51]).** *Let $f(u_{1},u_{2})$ be a real, concave and bounded-from-above function that is continuous and has continuous partial derivatives. Let $U_{1}$ and $U_{2}$ be two convex sets. Consider an optimization problem*

(A6) $$\begin{array}{c} \underset{u_{1}\in U_{1},\;u_{2}\in U_{2}}{\sup}f\left(u_{1},u_{2}\right)=f^{*}. \end{array}$$

*Denote $c_{2}\left(u_{1}\right)$ = $\sup_{u_{2}\in U_{2}}f\left(u_{1},u_{2}\right)$ and $c_{1}\left(u_{2}\right)$ = $\sup_{u_{1}\in U_{1}}f\left(u_{1},u_{2}\right)$. The alternating maximization algorithm is an iterative process where in each iteration k we maximize over one of the variables. Let $\left(u_{1}^{0},u_{2}^{0}\right)$ be an arbitrary starting point in $U_{1}\times U_{2}$. For k≥0 let $\left(u_{1}^{k},u_{2}^{k}\right)=\left(c_{1}\left(u_{2}^{k-1}\right),c_{2}\left(c_{1}\left(u_{2}^{k-1}\right)\right)\right)$ and let $f^{k}=f\left(u_{1}^{k},u_{2}^{k}\right)$. Assume that $c_{2}\left(u_{1}\right)\in U_{2}$ and $c_{1}\left(u_{2}\right)\in U_{1}$ are unique for all $u_{1}\in U_{1}$ and $u_{2}\in U_{2}$, then $\lim_{k\to \infty }f^{k}=f^{*}$.*

Let us reformulate (A5) according to the requirements of Lemma A1. First, we multiply the numerator and the denominator in the log by $\psi^{*}\left(\theta ,x\right)=\frac{\pi \left(\theta \right)p_{\theta }\left(x\right)}{q_{\pi }\left(x\right)}$. We attain

(A7) $$\begin{array}{c} \underset{\pi (\theta )}{\sup}\int_{\theta \in \Theta }\pi \left(\theta \right)\int_{x}p_{\theta }\left(x\right)\log\frac{\psi^{*}\left(\theta ,x\right)}{\pi (\theta )}\frac{p_{\theta }^{*}\left(x\right)}{p_{\theta }\left(x\right)}dxd\theta . \end{array}$$

Define the following maximization problem:

(A8) $$\begin{array}{c} \underset{\phi \left(\theta \right)\in A_{1}}{\sup}\underset{\psi \left(\theta ,x\right)\in A_{2}\left(x\right)}{\sup}\int_{\theta \in \Theta }\phi \left(\theta \right)\int_{x}p_{\theta }\left(x\right)\log\frac{\psi (\theta ,x)}{\phi (\theta )}\frac{p_{\theta }^{*}\left(x\right)}{p_{\theta }\left(x\right)}dxd\theta \end{array}$$

where $A_{1}=\left\{\phi \left(\theta \right)|\;\int_{\theta \in \Theta }\phi \left(\theta \right)d\theta =1,\;\phi \left(\theta \right)\geq 0\right\}$ and $A_{2}\left(x\right)=\left\{\psi \left(\theta ,x\right)|\;\int_{\theta \in \Theta }\psi \left(\theta ,x\right)d\theta =1,\;\psi \left(\theta ,x\right)\geq 0\right\}$ are convex sets. We now show that (A8) satisfies the conditions of the alternating maximization algorithm. First, we notice that our objective is real, concave, and bounded from above (as $F(\Theta ,\Theta_{r})\infty$). Lemmas A2 and A3 below show that there exists a unique maximum for every $\phi \left(\theta \right)\in A_{1}$ and $\psi \left(\theta ,x\right)\in A_{2}$, similarly to the Blahut–Arimoto algorithm for the channel capacity problem.

**Lemma** **A2.**

$$\underset{\psi \left(\theta ,x\right)\in A_{2}\left(x\right)}{\mathrm{argmax}}\int_{\theta \in \Theta }\phi \left(\theta \right)\int_{x}p_{\theta }\left(x\right)\log\frac{\psi (\theta ,x)}{\phi (\theta )}\frac{p_{\theta }^{*}\left(x\right)}{p_{\theta }\left(x\right)}dxd\theta =\frac{\phi \left(\theta \right)p_{\theta }\left(x\right)}{\int_{\theta^{\prime }\in \Theta }\phi \left(\theta^{\prime }\right)p_{\theta^{\prime }}\left(x\right)d\theta^{\prime }}.$$

**Proof.** Define $w\left(x\right)=\int_{\theta^{\prime }\in \Theta }\phi \left(\theta^{\prime }\right)p_{\theta^{\prime }}\left(x\right)d\theta^{\prime }$. Further, define $\psi^{\prime }\left(\theta ,x\right)=\frac{\phi \left(\theta \right)p_{\theta }\left(x\right)}{w(x)}$. We have that

(A9) $$\begin{array}{cc} \; & \int_{\theta \in \Theta }\phi \left(\theta \right)\int_{x}p_{\theta }\left(x\right)\log\frac{\psi^{\prime }\left(\theta ,x\right)}{\phi (\theta )}\frac{p_{\theta }^{*}\left(x\right)}{p_{\theta }\left(x\right)}dxd\theta -\int_{\theta \in \Theta }\phi \left(\theta \right)\int_{x}p_{\theta }\left(x\right)\log\frac{\psi (\theta ,x)}{\phi (\theta )}\frac{p_{\theta }^{*}\left(x\right)}{p_{\theta }\left(x\right)}dxd\theta =\\ \; & \int_{\theta \in \Theta }\phi \left(\theta \right)\int_{x}p_{\theta }\left(x\right)\log\frac{\psi^{\prime }\left(\theta ,x\right)}{\psi (\theta ,x)}dxd\theta =\int_{\theta \in \Theta }\int_{x}w\left(x\right)\psi^{\prime }\left(\theta ,x\right)\log\frac{\psi^{\prime }\left(\theta ,x\right)}{\psi (\theta ,x)}dxd\theta =\\ \; & \int_{x}w\left(x\right)D_{\mathrm{KL}}(\psi^{\prime }\left(\theta ,x\right)||\psi \left(\theta ,x\right))\geq 0 \end{array}$$

where the second equality follows from the definition of $\psi^{\prime }\left(\theta ,x\right)$ above.  □

**Lemma** **A3.**

$$\underset{\phi \left(\theta \right)\in A_{1}}{\mathrm{argmax}}\int_{\theta \in \Theta }\phi \left(\theta \right)\int_{x}p_{\theta }\left(x\right)\log\frac{\psi (\theta ,x)}{\phi (\theta )}\frac{p_{\theta }^{*}\left(x\right)}{p_{\theta }\left(x\right)}dxd\theta =\frac{\prod_{x}\tilde{\psi }{\left(\theta ,x\right)}^{p_{\theta }\left(x\right)}}{\int_{\theta^{\prime }\in \Theta }\prod_{x}\tilde{\psi }{\left(\theta^{\prime },x\right)}^{p_{\theta^{\prime }}\left(x\right)}d\theta^{\prime }}$$

*where $\tilde{\psi }\left(\theta ,x\right)=\psi \left(\theta ,x\right)\frac{p_{\theta }^{*}\left(x\right)}{p_{\theta }\left(x\right)}$*

**Proof.** We apply calculus of variations and attain the optimality condition. Define the Lagrangian as

(A10) $$\begin{array}{c} L=\int_{\theta \in \Theta }\phi \left(\theta \right)\int_{x}p_{\theta }\left(x\right)\log\frac{\psi (\theta ,x)}{\phi (\theta )}\frac{p_{\theta }^{*}\left(x\right)}{p_{\theta }\left(x\right)}dxd\theta -\lambda \left(\int_{\theta \in \Theta }\phi \left(\theta \right)d\theta -1\right)=\\ \int_{\theta \in \Theta }\phi \left(\theta \right)\left(\int_{x}p_{\theta }\left(x\right)\log\frac{\psi (\theta ,x)}{\phi (\theta )}\frac{p_{\theta }^{*}\left(x\right)}{p_{\theta }\left(x\right)}dx-\lambda \right)d\theta +\lambda \end{array}$$

Then, the Euler–Lagrange condition requires that the partial derivative of the integrand with respect to ϕ(θ) is zero. This yields

$$\int_{x}p_{\theta }\left(x\right)\left(\log\frac{\tilde{\psi }\left(\theta ,x\right)}{\phi (\theta )}-1\right)dx-\lambda =0$$

where $\tilde{\psi }\left(\theta ,x\right)=\psi (\theta ,x)\frac{p_{\theta }^{*}\left(x\right)}{p_{\theta }\left(x\right)}$. Therefore, $\phi \left(\theta \right)\propto \prod_{x}\tilde{\psi }{\left(\theta ,x\right)}^{p_{\theta }\left(x\right)}$ as desired. □

## Appendix C

Let X∼N(μ,Σ) be a *d*-dimensional Gaussian vector where μ is unknown and Σ is known. Let $x^{n}$ be a collection of *n* i.i.d. draws from *X*. Define a 100(1−α)% confidence region for μ as a collection $M_{r}=\left\{\mu |{\left(\bar{x}-\mu \right)}^{T}\Sigma^{-1}\left(\bar{x}-\mu \right)\leq \frac{1}{n}\chi_{d}^{2}\left(1-\alpha \right)\right\}$, as defined in Section 5. As previously established (and shown in [30]), our minimax problem is equivalent to a channel capacity problem X=M+Z where $M\in M_{r}$ and Z∼N(0,Σ), independent of *M*.

Let $\Sigma =USU^{T}$ be the singular value decomposition (SVD) of Σ and $A^{T}=S^{-0.5}U^{T}$ be the diagonalizing transformation of *Z*, such that $\mathrm{Cov}\left(S^{-0.5}U^{T}Z\right)=I_{d}$. Define $X^{\prime }=A^{T}X=A^{T}M+A^{T}Z$. Notice that $I\left(M;X\right)=I\left(A^{T}M;A^{T}X\right)$, given that *A* is invertible. Let us study $I(A^{T}M;A^{T}X)$. We have that $Z^{\prime }=A^{T}Z∼N\left(0,I_{d}\right)$ and

$${\left(A^{T}\bar{x}-A^{T}\mu \right)}^{T}\left(A^{T}\bar{x}-A^{T}\mu \right)={\left(\bar{x}-\mu \right)}^{T}AA^{T}\left(\bar{x}-\mu \right)={\left(\bar{x}-\mu \right)}^{T}\Sigma^{-1}\left(\bar{x}-\mu \right)\leq \frac{1}{n}\chi_{d}^{2}\left(1-\alpha \right).$$

This means that $\mu^{\prime }=A^{T}\mu$ satisfies $\mu^{\prime }\in M_{r}^{\prime }$ where

$$M_{r}^{\prime }=\left\{\mu^{\prime }|{\left(A^{T}\bar{x}-\mu^{\prime }\right)}^{T}\left(A^{T}\bar{x}-\mu^{\prime }\right)\leq \frac{1}{n}\chi_{d}^{2}\left(1-\alpha \right)\right\}.$$

Therefore, the channel $X^{\prime }=M^{\prime }+Z^{\prime }$ is again a standard amplitude constrained Gaussian channel, where the input is now a sphere around $A^{T}\bar{x}$, instead of $\bar{x}$.

Finally, we define $X^{\prime \prime }=X^{\prime }-A^{T}\bar{x}=M^{\prime }-A^{T}\bar{x}+Z^{\prime }$. Let $M^{\prime \prime }=M^{\prime }-A^{T}\bar{x}$ and $\mu^{\prime \prime }=\mu^{\prime }-A^{T}\bar{x}$. We have that $M^{\prime \prime }\in M_{r}^{\prime \prime }$ where $M_{r}^{\prime \prime }=\left\{\mu^{\prime }|\mu^{\prime \prime T}\mu^{\prime \prime }\leq \frac{1}{n}\chi_{d}^{2}\left(1-\alpha \right)\right\}.$ Further, we have that $I\left(M;X\right)=I\left(M^{\prime \prime };X^{\prime \prime }\right)$. This means that our original minimax problem is equivalent to a standard, centered, amplitude-constrained channel capacity problem.

## Appendix D

Let $q_{\pi }$ be convex combination of $V(P_{r})$. Assume that $q_{\pi }$ satisfies $D_{\mathrm{KL}}(p_{j}\left|\right|q_{\pi })=c$ for all $p_{j}\in V\left(P_{r}\right)$. Notice that every $p^{*}\in P_{r}$ can be described as a convex combination of the vertexes $V(P_{r})$ (since $P_{r}$ is a convex set). Therefore, we have that $D_{\mathrm{KL}}(p^{*}\left|\right|q_{\pi })=D_{\mathrm{KL}}(\sum a_{j}p_{j}\left|\right|q_{\pi })\leq \sum a_{j}D_{\mathrm{KL}}(p_{j}\left|\right|q_{\pi })=c$. This means that $q_{\pi }$ satisfies that optimality conditions over the set $P_{r}$. In other words, the solution to the minimax problem (2) over the confidence region $P_{r}$ is attained by solving the capacity-redundancy problem (3) over its finite set of vertexes $V(\Theta_{r})$.

## Appendix E. A Proof for Theorem 6

Let us first introduce the following Lemma.

**Lemma** **A4.** *Let q be a proper estimator. Then, $\Delta_{p}\left(n\right)\leq o\left(\frac{1}{n}\right).$*

**Proof.** Applying a simple mathematical induction,

(A11) $$\begin{array}{cc} ED_{\mathrm{KL}}(p||q\left(\cdot |x^{n}\right))= & \Delta_{p}\left(n\right)+ED_{\mathrm{KL}}(p||q\left(\cdot |x^{n+1}\right))=\\ \; & \Delta_{p}\left(n\right)+\Delta_{p}\left(n+1\right)+ED_{\mathrm{KL}}(p||q\left(\cdot |x^{n+2}\right))=\\ \; & \lim_{m\to \infty }\left(\sum_{k=n}^{m}\Delta_{p}\left(k\right)+ED_{\mathrm{KL}}(p||q\left(\cdot |x^{m}\right))\right)\infty \end{array}$$

where the finiteness is due to Condition *A*. Therefore, we necessarily have that the series $\sum_{k=n}^{m}\Delta_{p}\left(k\right)\infty$ converges. Conditions *B* and *C* state that $\Delta_{p}\left(k\right)$ is non-negative and monotonically non-increasing. Therefore, simple calculus shows that $\lim_{m\to \infty }m\Delta_{p}\left(m\right)=0$, which implies that $\Delta_{p}\left(m\right)\leq o\left(\frac{1}{m}\right)$. □

Now, define the *leave-one-out* model class as $P_{loo}={\left\{q\left(\cdot |x_{\left[-i\right]}^{n-1}\right)\right\}}_{i=1}^{n}$. We have that

(A12) $$\begin{array}{cc} \; & E\left(\min_{i}D_{\mathrm{KL}}\left(p||q\left(\cdot |x_{\left[-i\right]}^{n-1}\right)\right)\right)\leq \min_{i}E\left(D_{\mathrm{KL}}\left(p||q\left(\cdot |x_{\left[-i\right]}^{n-1}\right)\right)\right)=\\ \; & E\left(D_{\mathrm{KL}}\left(p||q\left(\cdot |x^{n-1}\right)\right)\right)=E\left(D_{\mathrm{KL}}\left(p||q\left(\cdot |x^{n}\right)\right)\right)+\Delta_{p}\left(n\right)\leq \\ \; & E\left(D_{\mathrm{KL}}\left(p||q\left(\cdot |x^{n}\right)\right)\right)+o\left(\frac{1}{n}\right) \end{array}$$

where the first inequality is due to Jensen inequality and the second inequality is due to Lemma A4.

## Appendix F

Define the expected convergence rate of an estimator $q(\cdot |x^{n})$ as

(A13) $$\begin{array}{c} \Delta_{p}\left(n\right)=E(D_{\mathrm{KL}}(p||q\left(\cdot |x^{n}\right))-D_{\mathrm{KL}}(p||q\left(\cdot |x^{n+1}\right))). \end{array}$$

The add-constant estimator follows $q\left(i|x^{n}\right)=q\left(n_{i}\right)=\frac{n_{i}+\beta }{n+m\beta }$ where β is the added constant. We have

(A14) $$\begin{array}{cc} ED_{\mathrm{KL}}(p||q\left(\cdot |x^{n}\right))= & E\sum_{i}p\left(i\right)\log\frac{p(i)}{q(n_{i})}=\\ \; & H\left(p\right)-\sum_{i}p\left(i\right)\left(E\log\left(n_{i}+\beta \right)-\log\left(n+m\beta \right)\right)=\\ \; & H\left(p\right)+\log\left(n+m\beta \right)-\sum p\left(i\right)E\log\left(n_{i}+\beta \right). \end{array}$$

For X∼Bin(n,θ) we have that $E\log\left(X+a\right)=\log\left(\theta n+a\right)-\frac{1-\theta }{2\theta n}+O\left(\frac{1}{n^{2}}\right)$. Further, $n_{i}=\sum_{j=1}^{n}𝟙\left\{x_{j}=1\right\}∼\mathrm{Bin}\left(n,p\left(i\right)\right)$. Therefore, $E\log\left(n_{i}+\beta \right)=\log\left(np\left(i\right)+\beta \right)-\frac{1-p(i)}{2np(i)}+O\left(\frac{1}{n^{2}}\right)$, leading to

(A15) $$\begin{array}{c} ED_{\mathrm{KL}}(p||q\left(\cdot |x^{n}\right))=H\left(p\right)+\log\left(n+m\beta \right)-\sum p\left(i\right)\log\left(np\left(i\right)+\beta \right)+\frac{m-1}{2n}+O\left(\frac{1}{n^{2}}\right). \end{array}$$

Plugging this result to (A13), we obtain

(A16) $$\begin{array}{cc} \Delta_{p}\left(n\right)= & \sum_{i}p\left(i\right)\log\left(\frac{n+m\beta }{np(i)+\beta }\cdot \frac{(n+1)p(i)+\beta }{(n+1)+m\beta }\right)+\frac{m-1}{2n(n+1)}+O\left(\frac{1}{n^{2}}\right)\\ \; & \sum_{i}p\left(i\right)\log\frac{(n+1)p(i)+\beta }{np(i)+\beta }+\frac{m-1}{2n(n+1)}+O\left(\frac{1}{n^{2}}\right)=\\ \; & -\sum_{i}p\left(i\right)\log\left(1-\frac{p(i)}{(n+1)p(i)+\beta }\right)+\frac{m-1}{2n(n+1)}+O\left(\frac{1}{n^{2}}\right)\leq \\ \; & \sum_{i}p\left(i\right)\frac{p(i)}{(n+1)p(i)+\beta }\cdot \frac{(n+1)p(i)+\beta }{\left(n+1)p(i)+\beta -p(i\right)}+\frac{m-1}{2n(n+1)}+O\left(\frac{1}{n^{2}}\right)=\\ \; & \sum_{i}\frac{p^{2}\left(i\right)}{np(i)+\beta }+\frac{m-1}{2n(n+1)}+O\left(\frac{1}{n^{2}}\right)\leq \sum_{i}\frac{p^{2}\left(i\right)}{np(i)}+\frac{m-1}{2n(n+1)}+O\left(\frac{1}{n^{2}}\right)=\\ \; & \frac{1}{n}+\frac{m-1}{2n(n+1)}+O\left(\frac{1}{n^{2}}\right) \end{array}$$

where the first inequality is due to n+mβn+1+mβ and the second inequality is $-\log\left(1-x\right)\leq \frac{x}{1-x}$ for all 0x1. Finally, we have

(A17) $$\begin{array}{cc} \; & E\left(\min_{i}D_{\mathrm{KL}}\left(p||q\left(\cdot |x_{\left[-i\right]}^{n-1}\right)\right)\right)\leq \min_{i}ED_{\mathrm{KL}}\left(p||q\left(\cdot |x_{\left[-i\right]}^{n-1}\right)\right)=\\ \; & ED_{\mathrm{KL}}\left(p||q\left(\cdot |x^{n-1}\right)\right)=\Delta_{p}\left(n-1\right)+ED_{\mathrm{KL}}(p||q\left(\cdot |x^{n}\right))\leq \\ \; & ED_{\mathrm{KL}}(p||q\left(\cdot |x^{n}\right))+\frac{1}{n-1}+\frac{m-1}{2n(n-1)}+O\left(\frac{1}{n^{2}}\right) \end{array}$$

where the first inequality is due to Jensen inequality, the first equality is since $x^{n}$ are i.i.d. draws, and the last inequality is due to (A16).

## Appendix G

One of the basic properties of most widely used finite alphabet estimators (MLE, add-constant, Good–Turing, and others) is the *natural* assumption; symbols that appear the same number of times are assigned the same probability estimate. This is not surprising, as it is easy to show that natural estimators maximize the expected estimation accuracy for a given set of samples (for example, see the work of Orlitsky and Suresh [41]). A natural estimator has k−1 degrees of freedom where *k* is the number of symbols with a unique frequency values $n_{i}$ (the cardinality of the *frequency of frequencies*, as denoted by Good [43]).

Our suggested estimation scheme is also in the natural domain. First, we choose a natural estimator (for example, Good–Turing). Then, given a set of samples $x^{n}$, we identify sets of symbols with the same frequency values $n_{i}$. Denote the number of unique frequencies as *k*. Define the mass of all the symbols with the same frequency *r* as $p^{mass}\left(r|x^{n}\right)=\sum_{n_{i}=r}p\left(i\right)$ for r=0,...,k. We construct a leave-one-out (loo) model class by first excluding a single sample at a time and applying our chosen estimator, $q(i|x^{n-1})$. Then, we set ${\hat{p}}_{loo}^{mass}\left(r|x^{n}\right)=\sum_{n_{i}=r}q\left(i|x^{n-1}\right)$. In words, the loo estimator of the $r^{th}$ mass is attained by excluding a single sample, applying the chosen estimator, and accumulating the estimates of all the symbols of the original mass *r*. Finally, the estimate of a single symbol in a mass simply ${\hat{p}}_{loo}\left(i|x^{n}\right)={\hat{p}}_{loo}^{mass}\left(n_{i}|x^{n}\right)/\sum_{j}1\left\{n_{j}=n_{i}\right\}$. Notice that the size of the model class is *k* (and not *n*).

For example, consider the set $x^{n}=\left\{1,1,1,2,2,2,3,3\right\}$ and a maximum likelihood estimator (which is simpler to illustrate then Good-Turing). We would like to find the loo class given $x^{n}$. We seek loo estimates for symbols that appear twice, $p^{mass}\left(r=2|x^{n}\right)$, for x=3, and three times, $p^{mass}\left(r=3|x^{n}\right)$, for x=1,2. We first remove the first symbol $x_{1}=1$, and get $q\left(X=1|x_{\left[-1\right]}^{n-1}\right)=q\left(X=3|x_{\left[-1\right]}^{n-1}\right)=2/7$. This leads to ${\hat{p}}_{loo}^{mass}\left(r=2|x^{n}\right)=2/7$ and ${\hat{p}}_{loo}\left(r=3|x^{n}\right)=5/7$. Notice that we get the same estimates as we remove $x_{4}=2$. Finally, by removing $x_{7}=3$ we attain $q\left(X=1|x_{\left[-7\right]}^{n-1}\right)=q\left(X=2|x_{\left[-7\right]}^{n-1}\right)=3/7$, leading to ${\hat{p}}_{loo}^{mass}\left(r=2|x^{n}\right)=1/7$ and ${\hat{p}}_{loo}^{mass}\left(r=3|x^{n}\right)=6/7$. Therefore, our corresponding loo model class ${\hat{p}}_{loo}$ consists of two estimates, ${\left[2.5/7,2.5/7,2/7\right]}^{T}$ and ${\left[3/7,3/7,1/7\right]}^{T}$ and its cardinality is k=2.
